# Significant Wave Height Estimation from Joint CYGNSS DDMA and LES Observations

**DOI:** 10.3390/s21186123

**Published:** 2021-09-12

**Authors:** Shuai Yang, Shuanggen Jin, Yan Jia, Mingda Ye

**Affiliations:** 1 Shanghai Astronomical Observatory, Chinese Academy of Sciences, Shanghai 200030, China; shuaiyang@shao.ac.cn (S.Y.); mdye@shao.ac.cn (M.Y.); 2 University of Chinese Academy of Sciences, Beijing 100049, China; 3 Department of Surveying and Geoinformatics, Nanjing University of Posts and Telecommunications, Nanjing 210023, China; jiayan@njupt.edu.cn

**Keywords:** GNSS-R, CYGNSS, SWH, DDMA, LES

## Abstract

The significant wave height (SWH) of oceans is the main parameter in describing the sea state, which has been widely used in the establishment of ocean process models and the field of navigation and transportation. However, traditional methods such as satellite radar altimeters and buoys cannot achieve SWH estimations with high spatial and temporal resolution. Recently, the spaceborne Global Navigation Satellite System reflectometry (GNSS-R) has provided an opportunity to estimate SWH with a rapid global coverage and high temporal resolution observations, particularly with the Cyclone Global Navigation Satellite System (CYGNSS) mission. In this paper, SWH was estimated using the polynomial function relationship between SWH from ERA5 and Delay-Doppler Map Average (DDMA) as well as Leading Edge Slope (LES) from CYGNSS data. Then, the SWH estimated from CYGNSS data was validated by ERA-Interim data, AVISO data, and buoy data. The results showed that the average correlation coefficient of CYGNSS SWH was 0.945, and the average RMSE was 0.257 m when compared to the ERA-Interim SWH data. The RMSE was 0.423 m and the correlation coefficient was 0.849 when compared with the AVISO SWH. The correlation coefficient with the buoy data was 0.907, and the RMSE was 0.247 m. This method can provide suitable SWH estimation data for ocean dynamics research and ocean environment prediction.

## 1. Introduction

The height and length of ocean waves vary randomly in time and space. This variance can be described by ocean wave spectra and parameters. Significant wave height (SWH) is defined as the average height of the highest one-third of waves in the wave spectrum [1], which is an important component of ocean wave parameters. High-quality SWH parameters are crucial to study climate change, ocean processes, and marine transportation. With the development of satellite technology, satellite observation has become an important approach for characterizing ocean waves. For instance, satellite radar altimeters have been used for global SWH estimations since 1970, such as GEOSAT (1985–1990), ERS-1 (1991–2000), Jason-2 (2008–2019), HY-2A (2011–present) [2], Jason-3 (2016–present), HY-2C (2020–present), and HY-2B (2018–present). Synthetic aperture radar (SAR) technology can also achieve SWH estimations [3]. The satellite observation techniques also can estimate sea level change [4] and coastal dynamic processes [5]. Currently, satellite altimeters can measure SWH with deviations of 3.8±4.0 cm [6], and SAR measurement can achieve a root mean square error of 0.32 m [3], but these techniques have long observation periods and cannot achieve high temporal resolution measurements. Though buoys can achieve high-temporal-resolution measurement, they cannot provide large-scale information on ocean waves [7]. Therefore, SWH estimations with high temporal and spatial resolution at a global scale are challenging.

Global Navigation Satellite System reflectometry (GNSS-R) is a passive remote sensing technique that uses GNSS signals reflected from Earth’s surface to obtain various information about the reflective surface characteristics [8,9]. GNSS-R technology has been used for monitoring sea surface height changes [10], soil moisture [11], snow accumulation [12], forests [13], ocean wind speed [14,15], and sea ice [16]. Yin et al. [17] confirmed that SWH was related to the significant coherence time of the interferometric complex field (ICF) [18], and showed that the SWH had an average deviation of 0.136 m and a root mean square error of 0.169 m. Alonso-Arroyo et al. [19] used interference pattern technology (IPT) to achieve SWH accuracy of 6 cm with a time resolution of 30 min. Wang et al. [20] proposed a new SWH retrieval method based on the expected values of the normalized waveform with an experimental average deviation of −0.06 m, a root mean square of 0.26 m, and a correlation coefficient of 0.93. Xu et al. [21] proposed a computational method for modeling derivative functions based on correlation functions (DCF) using airborne GNSS-R experiments. Ling et al. [22] proposed a computational method to estimate SWH using signal-to-noise ratio (SNR) modeling by reflected signals with an RMSE of 0.2431 m. The above inversion methods were based on ground-based or airborne GNSS-R equipment that cannot achieve large-range measurements, while the ICF and IPT methods are relatively complex. The UK-DMC satellite, which was launched by the United Kingdom in 2003, verified the feasibility of spaceborne GNSS-R technology [23]. The TechDemonsat-1 (TDS-1) satellite by Surrey Satellite Technology Ltd. launched in 2014 can generate Delay Doppler Map (DDM), which is an important data product for spaceborne GNSS-R technology [24]. In 2016, NASA successfully launched the spaceborne Cyclone Global Navigation Satellite System (CYGNSS) mission, which consists of eight small satellites with an orbital inclination of 35° and provides large-scale measurements with high spatial and temporal resolution [25]. This mission provided the opportunity to estimate SWH at a near-global scale through spaceborne GNSS-R observations. Peng et al. [26] first used the SNR of DDM data provided by CYGNSS to estimate SWH and verified the feasibility of SWH estimation from the spaceborne GNSS-R technique, while the SNR-based measurement method was derived from the X-band radar measurement formula. The Delay-Doppler Map Average (DDMA) and Leading Edge Slope (LES) generated from DDM data [27] can effectively reflect information on the physical parameters of the reflecting surface. Therefore, it is possible to estimate SWH using use the DDMA and LES data.

In this paper, a new SWH inversion method is presented using joint DDMA and LES measurements from CYGNSS. This method, based on CYGNSS, can achieve a larger observation range, better accuracy, and higher temporal resolution than previous ground- or air-based GNSS-R measurements. In the following, the SWH was estimated using DDMA, LES, and joint DDMA and LES, which were validated by ERA-Interim data, AVISO data, and buoy data. In Section 2, the CYGNSS data and SWH estimation methods are introduced. The results and accuracy analysis are presented in Section 3. Section 4 analyzes the error, and the conclusion is given in Section 5.

## 2. Data and Methods

### 2.1. CYGNSS Data

**Figure 1:**
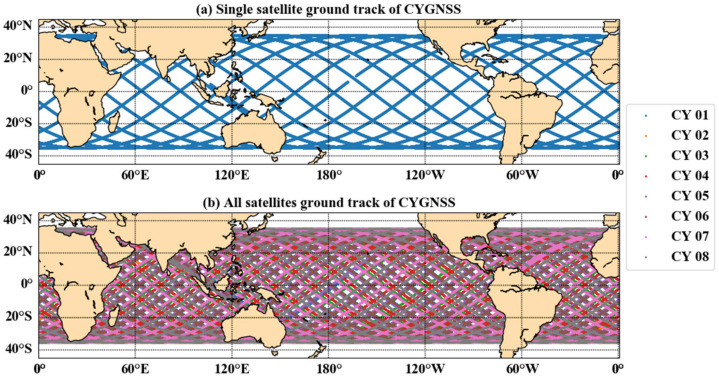
Ground track map for one CYGNSS satellite (**a**) and all CYGNSS satellites (**b**) on 6 June 2018.

Figure 1a shows the ground track of one CYGNSS satellite in one day, and Figure 1b presents the ground track of all CYGNSS satellites on the same day. The revisit time of CYGNSS is 3–7 h, which can estimate ocean parameters fast over a large area within the observation range. DDM is the main observation data of CYGNSS, which are 2D maps of scattered power from the reflecting surface. DDM is a function of the difference in propagation time and Doppler shift of the scattered signal relative to the direct signal. The maximum value of the DDM corresponds to the location of the specular reflection point (SP). DDMA and LES can be calculated and Equations (1)–(3) show the specific calculation process.

DDMA is the average scattered power computed over the specified delay/Doppler window of the DDM around the SP. Normally, the selection window is a 3 (Delay) × 5 (Doppler) matrix. The DDMA can be calculated by Equation (1), where $\Delta \tau =\tau_{m}-\tau_{1}$, $\Delta f=f_{n}-f_{1}$, $\bar{Y}(\tau_{m},f_{n},t_{i})$ is the scattered power value minus the noise at $\tau_{m}$ and $f_{n}$. LES is the slope of the leading edge of the integrated delay waveform (IDW) over a specified delay range (the normal choice is zero delays). LES can be calculated with Equations (2) and (3), wherein $\Delta \tau =\tau_{2}-\tau_{1}$ is the delay interval, α and c, respectively, represent the best-fit slope and intercept for linear fitting to the leading-edge region of the IDW leading-edge center point [27].


(1) $$\begin{array}{c} DDMA\left(\Delta \tau ,\Delta f,t_{i}\right)=\frac{1}{NM}\sum_{m=1}^{M}\sum_{n=1}^{N}\bar{Y}\left(\Delta \tau_{m},f_{n},t_{i}\right) \end{array}$$

(2) $$\begin{array}{c} IDW\left(\Delta \tau_{k},\Delta f,t_{i}\right)=\frac{1}{N}\sum_{n=1}^{N}\bar{Y}\left(\Delta \tau_{k},f_{n},t_{i}\right) \end{array}$$

(3) $$\begin{array}{c} LES\left(\Delta \tau ,\Delta f,t_{i}\right)=\underset{\alpha ,c}{\arg\min}\{\left[\sum_{k=1}^{2}IDW\left(\Delta \tau ,\Delta f,t_{i}\right)-\left(\alpha \tau_{k}+c\right){]}^{2}\right\} \end{array}$$

Here DDMA, LES, and latitude and longitude values of SP were chosen as parameters. To obtain high-quality observational data, the CYGNSS Level 1 version 2.1 data was filtered. The filter conditions were as follows:

(1) Some invalid data were eliminated through the quality control flags for CYGNSS data;
(2) Data with specular reflection points more than 25 km far away from the land were selected to reduce the modeling error;
(3) Observation data range was defined as 38° N–38° S in the latitude and 0–360° in the longitude.

### 2.2. Model Data

**Figure 2:**
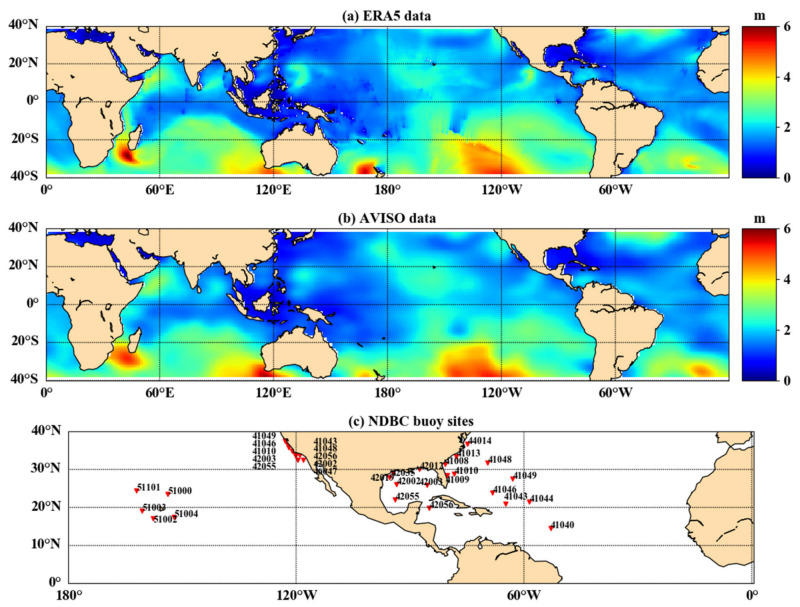
Distribution of ERA5 data on 6 June 2018 (**a**), AVISO data on 6 June 2018 (**b**), and selected NDBC buoy sites (**c**).

SWH data from the fifth-generation reanalysis (ERA5) [28] dataset published by the European Center for Medium Range Weather Forecasts (ECMWF) were applied as the true values. ERA5 is an upgrade to ERA-Interim, which was improved temporal resolution from 6 h to 1 h, compared to ERA-Interim, and existing studies have shown that results based on ERA5 data are superior to those based on ERA-Interim data [29,30]. The range of the selected ERA5 SWH data was consistent with CYGNSS data, with a spatial range of 38° N to 38° S, 180° W to 180° E, and a spatial resolution of $0.5^{{}^{\circ}}\times 0.5^{{}^{\circ}}$. The ERA-Interim data and the AVISO data are used as comparison at a large scale. However, the AVISO dataset has a spatial resolution of $1^{{}^{\circ}}\times 1^{{}^{\circ}}$ and a temporal resolution of one day. Thus, the estimation results from the CYGNSS data need to be matched with AVISO data. Buoy observations from the National Data Buoy Center (NDBC) were selected for comparison. In order to match CYGNSS observations, the buoy should be far from land (>25 km) with spatially ranging between 38° N and 38° S, and working properly in 2018. The coverage of the selected ERA5 data and AVISO data on 6 June 2018 is shown in Figure 2. The distribution of the selected buoys is shown in Figure 2c.

### 2.3. SWH Estimation

Different sea conditions cause different degrees of reflection and scattering of electromagnetic signals on the sea surface. The GNSS signal is reflected by the sea level and received by the reflector antenna of the CYGNSS satellite. As a result, the DDM based on the difference between the reflected and direct signal can reflect various physical parameters of the sea surface. To estimate SWH using CYGNSS data, it is necessary to establish the correlation between the observed data and the SWH data. Since the traditional Pearson correlation coefficient (*R*) is only applicable to linear correlation analysis, this paper introduced the maximal information coefficient (MIC) for nonlinear correlation analysis. MIC is a correlation analysis method [31], which uses an unequal interval optimization method and has the advantages of universality and equivalence. The MIC value ranges from 0 to 1. This parameter can effectively reflect the degree of correlation between the two variables [32,33], which is calculated from Equation (4). In Equation (4), x and y are arbitrary variables, P(x,y) is the joint probability density function, P(x) and P(y) are the edge density function, m and n are the given frame size, and B is taken to the 0.6 power of the data.


(4) $$\begin{array}{c} MIC\left(x,y\right)=\max_{m\times n<B}\frac{\sum_{x}\sum_{y}P\left(x,y\right)\log_{2}\frac{P(x,y)}{P(x)P(y)}}{\log_{2}\left(\min\left(x,y\right)\right)} \end{array}$$

**Figure 3:**
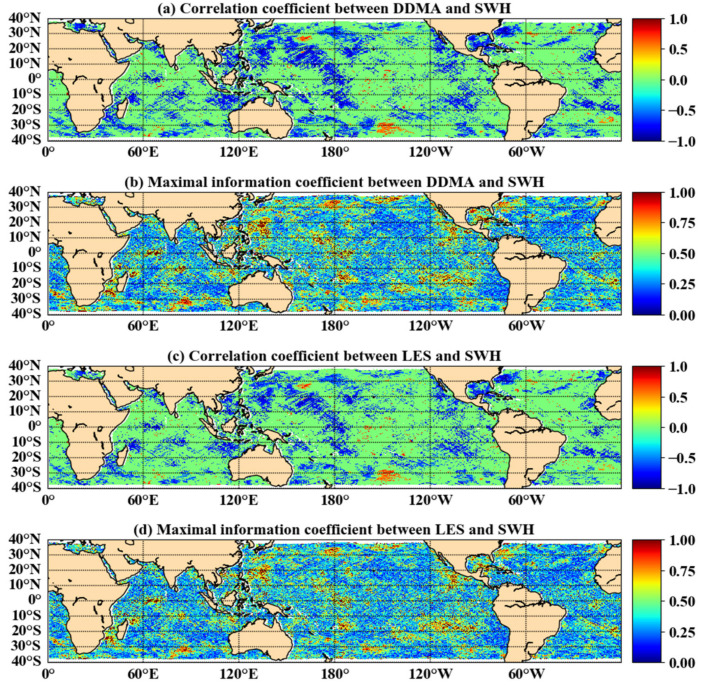
Distribution of correlation coefficients and maximal information coefficients between DDMA/LES and SWH.

Figure 3 shows the linear and nonlinear relationship between DDMA/LES and SWH within the same time scale (7 days), where the mean absolute value of *R* between DDMA and SWH was 0.175 and the average value of MIC was 0.417, while the mean absolute value of *R* between LES and SWH was 0.147 and the value of MIC was 0.401. Figure 3a,c show that the absolute value of *R* between DDMA or LES and SWH was mostly close to zero without a clear linear correlation. The distribution of the MIC parameters of the SWH is shown in Figure 3b,d. The MIC values are greater than 0.25 in most areas, showing that DDMA and LES have a good nonlinear correlation with SWH. Therefore, it is feasible to estimate SWH by establishing a functional expression between DDMA/LES and SWH.

Because of the complexity and variability of the marine environment, this study divided the observations according to a $0.5^{{}^{\circ}}\times 0.5^{{}^{\circ}}$ grid. While it is evident from Figure 3 that DDMA/LES had a good nonlinear correlation with SWH, the specific functional form was unknown. Hence, the n-order polynomial (n ranging from 2 to 10) in Equation (5) was chosen for parameter fitting in this paper, where x represents the DDMA or LES value, ω is the fitting coefficient, and y is the SWH data provided from the ERA5 dataset.


(5) $$\begin{array}{c} y\left(x,\omega \right)=\omega_{0}x^{0}+\omega_{1}x^{1}+\omega_{2}x^{2}+\ldots +\omega_{n}x^{n}=\sum_{i=0}^{n}\omega_{i}x^{i} \end{array}$$

**Figure 4:**
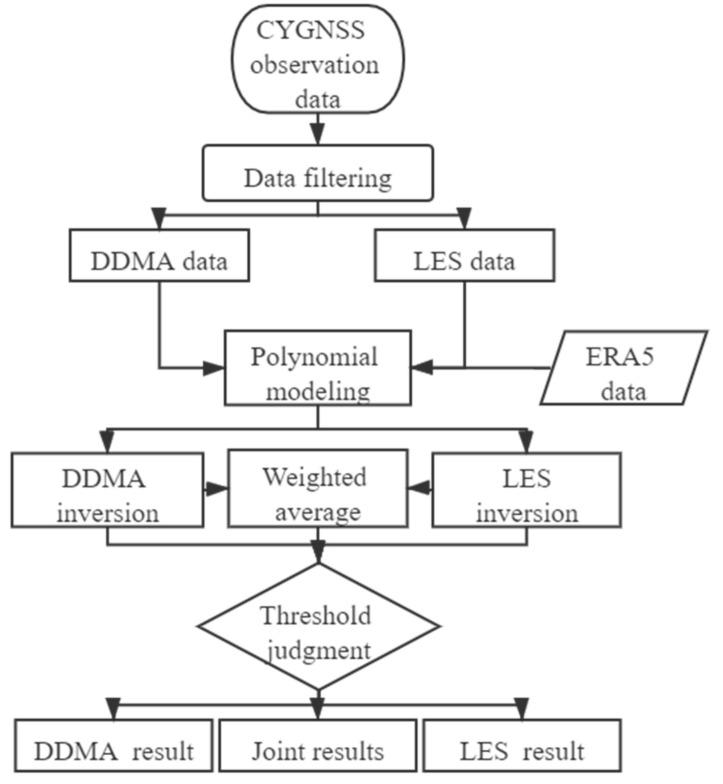
Flowchart diagram of the three inversion methods.

Based on the n-order polynomial form of Equation (5), three SWH inversion methods are proposed in this paper, namely: (1) an n-order inversion method based on DDMA (DDMA); (2) an n-order inversion method based on LES (LES); and (3) an n-order joint inversion method from both DDMA and LES (DDMA–LES). The calculation process of the three methods is shown in Figure 4. The filtering conditions for CYGNSS data are the same as those calculated for CYGNSS Level 2 data, which is DDMA from 0–1000 and LES from −300–1700. The weighted average is based on the degree of fitting between DDMA and LES, and the threshold of the data is set as the maximum value of SWH in the training data for subsequent accuracy comparisons.

### 2.4. Data Comparison Method

To quantify the various accuracy indicators in the data processing, four evaluation indicators were used: mean deviation (Bias), root mean square error(RMSE), Pearson correlation coefficient (*R*), and mean absolute error (MAE). In the following Equations (6)–(9), $x_{i}$ is an estimated value from CYGNSS data; $y_{i}$ represents ERA5 data, ERA-Interim data, AVISO data, or buoy observation value; $\bar{x}$ and $\bar{y}$ represent the average of x and y; and N is the amount of data involved in the calculation.


(6) $$\begin{array}{c} RMSE=\sqrt{\frac{{\sum_{i=1}^{N}\left(x_{i}-y_{i}\right)}^{2}}{N}} \end{array}$$

(7) $$\begin{array}{c} Bias=\frac{\sum_{i=1}^{N}\left(x_{i}-y_{i}\right)}{N} \end{array}$$

(8) $$\begin{array}{c} R=\frac{\sum_{i=1}^{N}\left(x_{i}-\bar{x}\right)\left(y_{i}-\bar{y}\right)}{\sqrt{\sum_{i=1}^{N}{\left(x_{i}-\bar{x}\right)}^{2}{\left(y_{i}-\bar{y}\right)}^{2}}} \end{array}$$

(9) $$\begin{array}{c} MAE=\frac{\sum_{i=1}^{N}\left|x_{i}-y_{i}\right|}{N} \end{array}$$

## 3. Results and Evaluation

### 3.1. SWH from CYGNSS

**Figure 5:**
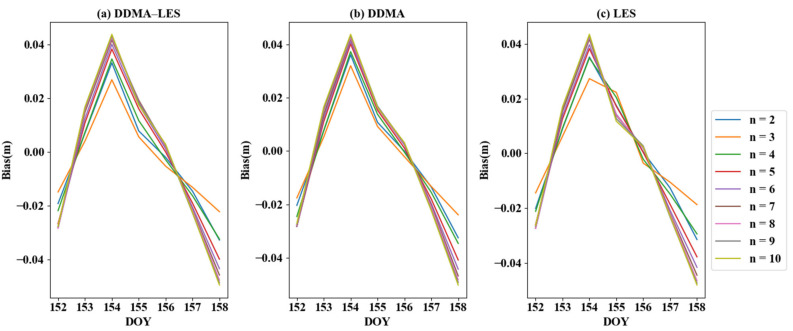
The bias of different order functions with three inversion methods.

Based on the three methods presented in Section 2.3, the data from 152 to 158 days of the year (DOY) in 2018 were chosen for case studies. Fitting was carried out using a polynomial function, where n ranges from 2 to 10. Figure 5 is a schematic representation of the bias between the CYGNSS inversion results and the ERA5 data. All three methods have large bias values for each order function on DOY 154 and 158, although these values are still within a small deviation range (±0.04 m). When the value of n is 3, the deviation of the error reached its minimum. The bias of the joint inversion results based on DDMA and LES was 0.0382 m. The mean bias based on DDMA inversion was 0.0398 m, and the average deviation based on LES inversion was 0.0383 m. The mean bias distributions of the three inversion methods were highly similar.

**Figure 6:**
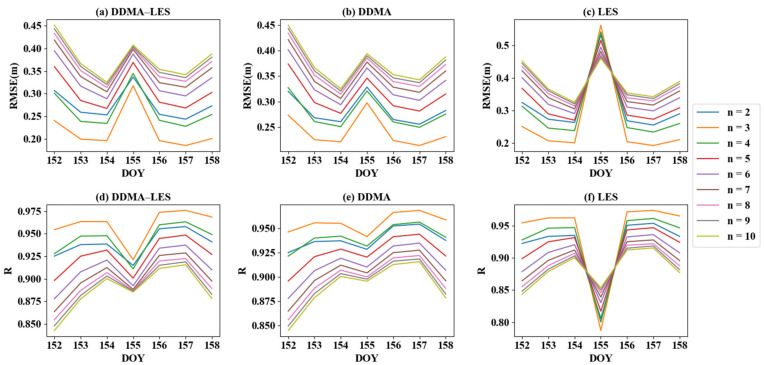
*R* and RMSE values of different order functions from three inversion methods.

Figure 6a–c shows the RMSE distributions for the three inversion methods. The results of the LES inversion method have a very large deviation on the DOY 155. The DDMA and DDMA–LES methods also yielded a peak on DOY 155, however, the amplitude was relatively small. When n was set to 3, all three inversion methods had excellent RMSE values. The average RMSE was 0.2778 m based on the DDMA–LES method, 0.2852 m based on DDMA inversion, and 0.2812 m based on LES inversion.

*R* distributions for the three inversion methods are shown in Figure 6d–f. The LES inversion method shows that the *R* distribution was symmetrical to the RMSE distribution. The LES method also had a large *R* bias on DOY 155, therefore, the results of the LES method were poorer on DOY 155. The DDMA and DDMA–LES methods showed relatively little fluctuation on DOY 155. It can be seen from Figure 6 that there was a good correlation between RMSE and *R*. The average *R* was 0.9250 based on the DDMA–LES method, 0.9226 based on DDMA inversion, and 0.9243 based on LES inversion. The above analysis of bias, RMSE, and *R* demonstrated which order of polynomial was best and that the joint inversion method using DDMA and LES was the best.

**Figure 7:**
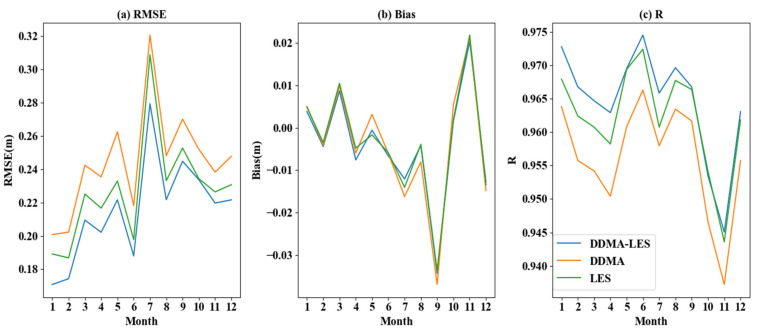
Bias, R, and RMSE value of different functions of three inversion methods.

To verify the quality of the three inversion methods and improve generality and reliability, an analysis of one modeling cycle is selected for each month of 2018. As shown in Figure 7, the DDMA–LES method consistently provided good stability and accuracy. The mean bias of the DDMA–LES method was −0.0040 m, the mean RMSE was 0.2156 m, and the mean *R* value was 0.9646. The mean bias of the DDMA method was −0.0040 m, the mean RMSE was 0.2449 m, and the mean *R* value was 0.9562. The mean bias based on LES inversion was −0.0035 m, the mean RMSE was 0.2279 m, and the mean *R* value was 0.9621. The joint inversion of DDMA and LES based on third-order polynomial functions had a slightly larger average bias than the DDMA and LES inversions alone, but with better RMSE and *R*. Therefore, the joint inversion method was the best of the three methods.

### 3.2. Comparing with ERA-Interim SWH Data

**Figure 8:**
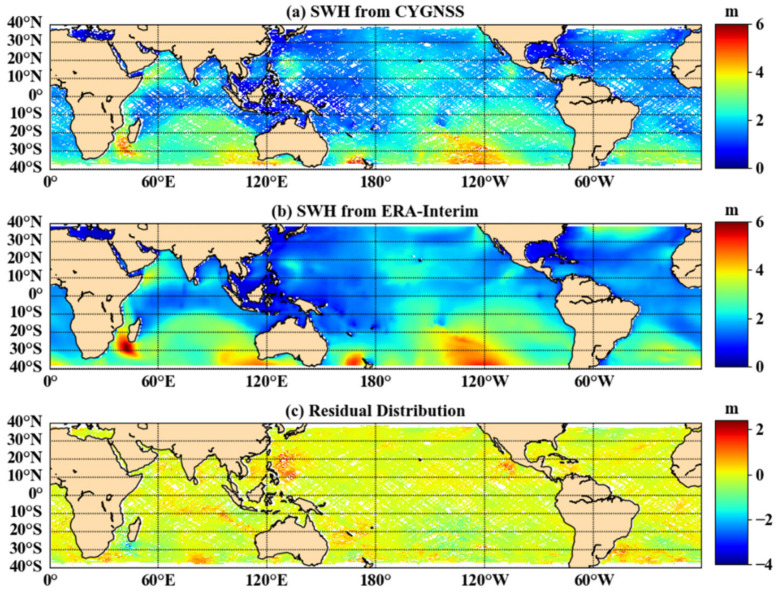
The difference between the CYGNSS estimated SWH and ERA-Interim (DOY 157, 2018).

Following the analysis in Section 3.1, the joint DDMA and LES inversion method based on third-order polynomial functions was selected for subsequent accuracy verification and error analysis in this paper. To ensure consistency of data evaluation criteria in this paper, the ERA-Interim data needed to be converted from a temporal resolution of 6 h to one of one day. Figure 8a shows the SWH results of the joint inversion, Figure 8b shows the ERA-Interim data, and Figure 8c shows the distribution of deviations between the inversion results and the ERA-Interim data. The white areas in Figure 8 are caused by missing or exceeded thresholds in the observed data. Most of the deviations were concentrated around zero, as shown in Figure 8c, but there was a region of larger deviations in the marginal region and near the Philippines. There are two reasons for this phenomenon: 1. the margin region is near 40° S, where there are fewer valid observations, resulting in a poor fit to the model; 2. the dense distribution of islands in the Philippines leads to poor inversion results based on CYGNSS data. Table 1 shows the results of the joint inversion with reference values for various evaluation indicators in 2018. The average bias was −0.008 m, the mean RMSE was 0.257 m, and the average *R* is 0.945. Thus, the results of the joint inversion with DDMA and LES were highly correlated with the reference values and gave a strong indication of the distribution of SWH.

### 3.3. Comparison with AVISO SWH Data

**Figure 9:**
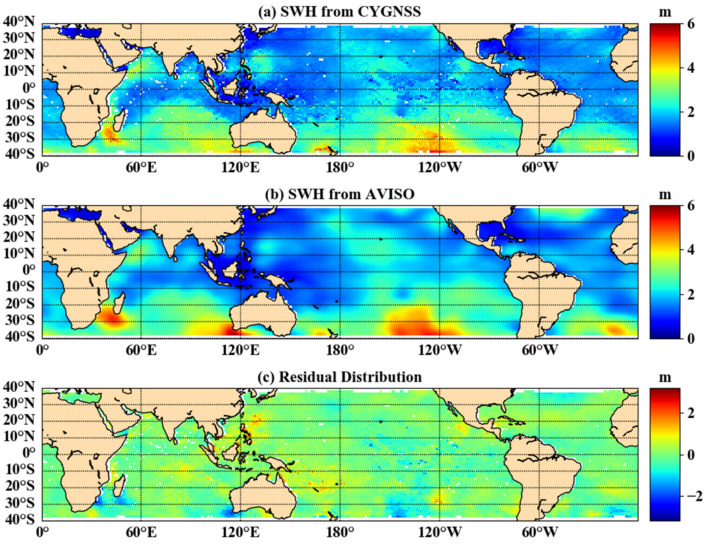
The difference between the CYGNSS inversion result of SWH and AVISO (DOY 157, 2018).

The ERA5 dataset provided by the ECMWF was used as the reference true value to build a polynomial model of SWH based on the joint inversion of DDMA and LES. Thus, the temporal resolution of the data was 1 h, and the spatial resolution was $0.5^{{}^{\circ}}\times 0.5^{{}^{\circ}}$. To compare and analyze data provided by AVISO, with a temporal resolution of 1 day and a spatial resolution of $1^{{}^{\circ}}\times 1^{{}^{\circ}}$, the inversion results were transformed into a gridded product with the same resolution. The SWH distribution of the joint inversion method is shown in Figure 9a, the AVISO data distribution is in Figure 9b, and the deviation distribution of the inversion results from the AVISO data is in Figure 9c. Figure 9 shows that the deviation values were mainly concentrated at −0.8 m, and the larger areas of deviation were similar to those in Section 3.2. Table 2 shows the difference evaluation index between the joint inversion results and AVISO data in 2018. The average bias was 0.008 m, the average RMSE was 0.423 m, and the average *R* was 0.849. Comparison of the AVISO data with the ERA-Interim data shows that the monthly average bias values in 2018 were of opposite sign, and the AVISO data had poor RMSE and *R* values. Firstly, the SWH inversion from CYGNSS data uses the ERA5 data as the reference true value, and therefore, the difference between the ERA5 data and the AVISO data results in poor RMSE and *R* values. Secondly, the reclassification of the inversion causes a change in the deviation values.

### 3.4. Comparison with Buoy Data

**Figure 10:**
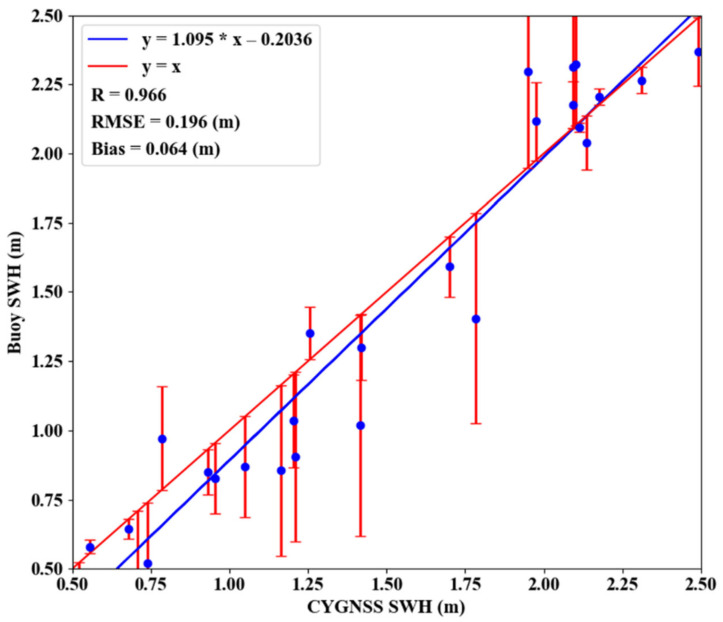
The difference between the CYGNSS inversion result of SWH and Buoy (DOY 157, 2018).

Buoy data is an important component of SWH measurements in addition to the two types of data mentioned above. To ensure the consistency of the accuracy evaluation, the following treatment was applied to the buoy data: buoy coordinates were converted to a grid of $0.5^{{}^{\circ}}\times 0.5^{{}^{\circ}}$ distribution, the error value in the buoy data was deducted and the observations were averaged over the day. Figure 10 shows the distribution and correlation between buoy data and joint inversion results. The linear relationship between the inversion results and the buoy value can be represented as y=1.095×x−0.2036. The error bar distribution in Figure 10 shows that the deviation between the inversion results and the buoy observations did not vary with the SWH value. Table 3 shows the evaluation index of the inversion results and the buoy data. The average bias was 0.074 m, the average RMSE was 0.247 m and the average *R* was 0.907. Therefore, the joint CYGNSS inversion results were correlated well with the buoy observations.

## 4. Error Analysis

In Section 3, the results of the joint inversion method of DDMA and LES based on the third-order polynomial are presented and compared. The results were demonstrated a high accuracy SWH estimation through comparative analysis with ERA-Interim data, AVISO data, and buoy data. The CYGNSS data can estimate SWH with high spatial resolution, which is beneficial to the study of small-scale SWH variations.

**Figure 11:**
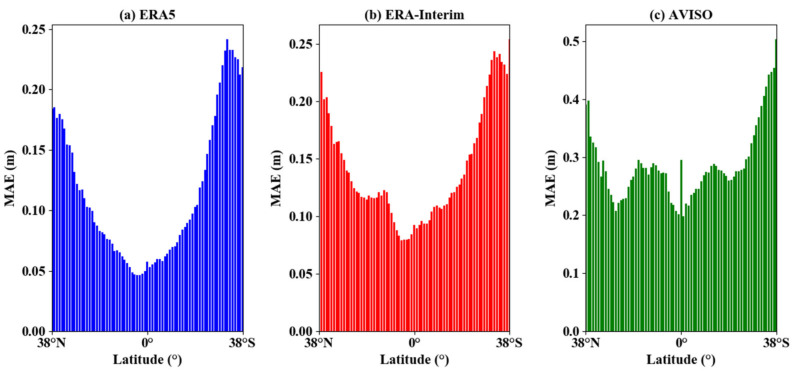
Distribution of MAE with the latitude for different data.

However, the errors in the inverse model shift with the latitude. Figure 11 shows the distribution of MAE values for the CYGNSS inversion results with ERA5, ERA-Interim, and AVISO data at 1° interval. It can be found that the MAE values were smaller in lower-latitude areas, and the error values were higher in high-latitude areas. The difference between the AVISO data and the ERA5 data resulted in a large bias in the error distribution at lower latitudes as well. The data from ERA-Interim fit well with the data from ERA5. Since the MAE was within 0.2 m in regard to the ERA5 dataset, which was lower than the RMSE of the model, it did not have a significant impact on the model. The bias in the ERA5 data and ERA-Interim data was raised because the model fitting used the small amount of CYGNSS data at high latitudes, and the large bias in the AVISO data was probably due to the regridding of the model data from $0.5^{{}^{\circ}}\times 0.5^{{}^{\circ}}$ to $1^{{}^{\circ}}\times 1^{{}^{\circ}}$.

**Figure 12:**
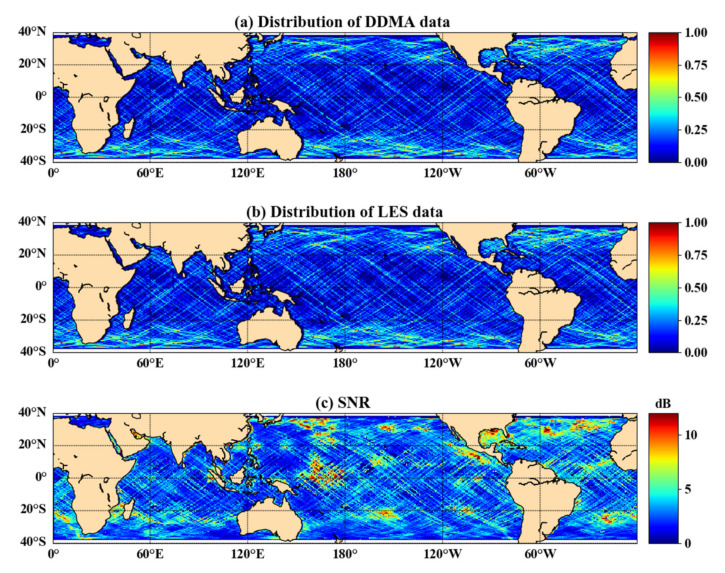
(**a**) Normalized distribution of DDMA data volumes; (**b**) normalized distribution of LES data volumes; and (**c**) SNR distribution of CYGNSS reflection points (DOY 215, 2019).

Furthermore, the SNR of CYGNSS sea surface observation data was poor. Figure 12c shows the SNR distribution of sea surface reflection points during the day. The maximum SNR during the day at sea was only 12 dB, and most areas are between 0–4 dB. The lower SNR affects data quality and model accuracy. The distribution of the normalized DDMA and LES data is shown in Figure 12a,b. Figure 12 shows that the amount of data was not necessarily large where the SNR was high.

To further improve the SWH accuracy of the joint DDMA and LES inversion, the high SNR data of the CYGNSS observations should be used. However, an excessively high SNR would reduce the number of observations, which will degrade the inversion accuracy. 

## 5. Conclusions

In this paper, three methods were proposed to estimate SWH from CYGNSS data based on polynomial function models. The accuracy and stability of the three inversion methods at different n-orders were analyzed from 2018 DOY 152 to 158. The results showed that the accuracy and stability of the third-order polynomial function were the best. Through analysis of the data extracted for each month of 2018, the joint inversion results based on DDMA and LES gave an average bias of −0.0040 m, an average RMSE value of 0.2156 m, and an average *R* of 0.9646. The results showed that the accuracy of the joint inversion based on the DDMA and LES methods was the best.

The accuracy and reliability of the method were further analyzed by comparing the joint inversion results with ERA-Interim data, AVISO data, and NDBC buoy data. The mean bias between the joint inversion result and the ERA-Interim data was −0.008 m, the mean RMSE was 0.257 m, and the mean *R* value was 0.945. Because of the differences between the ERA5 data and the AVISO data, the *R* values between the inversion results and the AVISO data were minimal, but the correlation was high. The mean bias was 0.008 m, the mean RMSE was 0.423 m and the mean *R* value was 0.849. The average bias of the CYGNSS inversion results and buoy data was 0.074 m, the average RMSE was 0.247 m, and the average *R* was 0.907. Furthermore, the difference between the joint inversion result and the buoy data did not change with the SWH. Therefore, the joint inversion method from CYGNSS DDMA and LES can well estimate SWH with high accuracy and realize the rapid global SWH estimation at a large scale, which provides a new data source for ocean dynamics research.

## Figures and Tables

**Table 1:** Comparison of CYGNSS results and ERA-Interim data.

DOY	23	39	70	111	143	157	185	226	247	287	306	342
**RMSE (m)**	0.246	0.227	0.246	0.233	0.329	0.247	0.304	0.257	0.267	0.245	0.242	0.240
**Bias (m)**	−0.016	−0.014	0.007	−0.040	0.009	−0.024	0.005	−0.014	−0.023	−0.005	0.023	−0.006
**R**	0.938	0.937	0.946	0.950	0.927	0.956	0.954	0.957	0.956	0.943	0.926	0.952

**Table 2:** Comparison of CYGNSS results and AVISO data.

DOY	23	39	70	111	143	157	185	226	247	287	306	342
**RMSE (m)**	0.391	0.400	0.438	0.425	0.488	0.386	0.489	0.457	0.427	0.412	0.331	0.429
**Bias (m)**	0.024	−0.035	−0.030	−0.025	0.037	−0.003	0.064	0.069	0.005	−0.025	0.011	0.004
**R**	0.844	0.808	0.845	0.817	0.842	0.889	0.858	0.863	0.863	0.836	0.864	0.858

**Table 3:** Comparison of CYGNSS results and buoy data.

DOY	23	39	70	111	143	157	185	226	247	287	306	342
**RMSE (m)**	0.331	0.281	0.310	0.184	0.308	0.196	0.197	0.164	0.238	0.206	0.219	0.328
**Bias (m)**	0.042	0.077	0.136	0.004	0.159	0.064	0.035	0.072	0.074	0.133	0.061	0.032
**R**	0.932	0.915	0.873	0.945	0.855	0.966	0.943	0.958	0.810	0.910	0.894	0.883
